# A *Drosophila* model of mitochondrial disease phenotypic heterogeneity

**DOI:** 10.1242/bio.060278

**Published:** 2024-02-28

**Authors:** Lucy Granat, Debbra Y. Knorr, Daniel C. Ranson, Ram Prosad Chakrabarty, Navdeep S. Chandel, Joseph M. Bateman

**Affiliations:** ^1^ Maurice Wohl Clinical Neuroscience Institute, King's College London, 5 Cutcombe Road, London SE5 9RX, UK; ^2^ Department of Medicine, Biochemistry and Molecular Genetics, Northwestern University Feinberg School of Medicine, Chicago, IL 60611, USA

**Keywords:** Mitochondria, Phenotypic heterogeneity, Signalling, Complex I deficiency, Metabolism

## Abstract

Mutations in genes that affect mitochondrial function cause primary mitochondrial diseases. Mitochondrial diseases are highly heterogeneous and even patients with the same mitochondrial disease can exhibit broad phenotypic heterogeneity, which is poorly understood. Mutations in subunits of mitochondrial respiratory complex I cause complex I deficiency, which can result in severe neurological symptoms and death in infancy. However, some complex I deficiency patients present with much milder symptoms. The most common nuclear gene mutated in complex I deficiency is the highly conserved core subunit NDUFS1. To model the phenotypic heterogeneity in complex I deficiency, we used RNAi lines targeting the *Drosophila* NDUFS1 homolog ND-75 with different efficiencies. Strong knockdown of ND-75 in *Drosophila* neurons resulted in severe behavioural phenotypes, reduced lifespan, altered mitochondrial morphology, reduced endoplasmic reticulum (ER)-mitochondria contacts and activation of the unfolded protein response (UPR). By contrast, weak ND-75 knockdown caused much milder behavioural phenotypes and changes in mitochondrial morphology. Moreover, weak ND-75 did not alter ER-mitochondria contacts or activate the UPR. Weak and strong ND-75 knockdown resulted in overlapping but distinct transcriptional responses in the brain, with weak knockdown specifically affecting proteosome activity and immune response genes. Metabolism was also differentially affected by weak and strong ND-75 knockdown including gamma-aminobutyric acid (GABA) levels, which may contribute to neuronal dysfunction in ND-75 knockdown flies. Several metabolic processes were only affected by strong ND-75 knockdown including the pentose phosphate pathway and the metabolite 2-hydroxyglutarate (2-HG), suggesting 2-HG as a candidate biomarker of severe neurological mitochondrial disease. Thus, our *Drosophila* model provides the means to dissect the mechanisms underlying phenotypic heterogeneity in mitochondrial disease.

## Teaser

**Summary:** This study uses *Drosophila* to model the symptomatic variability in mitochondrial disease and provides mechanistic insight into the effects of weak and strong complex I deficiency in the brain.

## INTRODUCTION

Primary mitochondrial diseases are a broad spectrum of disorders characterised by defects in mitochondrial oxidative phosphorylation (OXPHOS). Primary mitochondrial diseases are caused by inherited or spontaneous mitochondrial DNA (mtDNA) or nuclear DNA mutations in genes required for mitochondrial function (Russell et al., 2020). These include genes encoding OXPHOS subunits, enzymes required for mtDNA maintenance, and enzymes involved in regulating mitochondrial gene expression (Gorman et al., 2016).

Mitochondrial diseases can arise both during childhood and adulthood, although this is often dictated by the type of genetic mutation involved. Early-onset mitochondrial diseases, where symptoms develop during infancy or childhood, are often caused by autosomal recessive mutations (Skladal et al., 2003). Adult-onset mitochondrial diseases are largely caused by mtDNA mutations and are generally less severe than those that are early-onset (Gorman et al., 2015). Both early-onset and adult-onset mitochondrial diseases are commonly associated with the development of neurological symptoms, which is likely due to the high metabolic demand of the central nervous system (CNS).

The prevalence of primary mitochondrial diseases is predicted to be ∼1 in 7634 at birth, and ∼1 in 4300 by adulthood (Skladal et al., 2003; Gorman et al., 2015). As a collective and individually, primary mitochondrial diseases are highly heterogeneous; the age of onset, tissue-specificity, and symptom severity can all vary depending on the individual and gene affected. Mitochondrial diseases are classified based on clinical symptoms, however, the genetic and phenotypic heterogeneity within some of these diseases can make diagnosis very challenging, and often patients do not fit within the defined criteria (Gorman et al., 2016; Finsterer et al., 2018). The underlying reasons for phenotypic heterogeneity within individual primary mitochondrial diseases are not well understood, which makes it challenging to identify common and distinct pathogenic mechanisms. Furthermore, our lack of knowledge surrounding the pathogenesis of mitochondrial diseases has made it difficult to develop therapies.

Complex I deficiency is the most common childhood mitochondrial disease and is caused by mutations in genes encoding complex I structural or assembly components (Fassone and Rahman, 2012). Complex I deficiency encompasses a broad spectrum of disorders with considerable phenotypic heterogeneity (Koene et al., 2012; Bjorkman et al., 2015). Mutations in complex I subunits can also cause Leigh syndrome, a progressive neurological disorder characterised by severe psychomotor regression, seizures, dystonia, dysphagia, spasticity, developmental delay and optic atrophy, alongside the presence of symmetrical bilateral lesions within the brainstem and basal ganglia (Lake et al., 2016). NDUFS1 is the most commonly mutated nuclear gene causing complex I deficiency (Fassone and Rahman, 2012). NDUFS1 is an [Fe-S] cluster containing subunit involved in electron transfer that lies within the N module of complex I (Wirth et al., 2016). Mutations in NDUFS1 typically cause severe and rapidly progressive leukoencephalopathy and death within the first 2 years of life (Kashani et al., 2014). However, phenotypic heterogeneity has been reported in NDUFS1 complex I deficiency, including several paediatric patients with much more mild symptoms (Hoefs et al., 2010; Ferreira et al., 2011; Fassone and Rahman, 2012; Kashani et al., 2014; Bjorkman et al., 2015).

Animal models of mitochondrial disease have been highly successful in revealing underlying mechanisms and identifying potential disease modifying therapies (Duncan and Bateman, 2016; Hunt and Bateman, 2018; Chen et al., 2019; Granat et al., 2020; van de Wal et al., 2022). Mirroring the diversity of mitochondrial diseases, animal models can vary greatly in the range and severity of phenotypes. However, none of these models have recapitulated the phenotypic heterogeneity observed within mitochondrial disease caused by mutations in the same gene. We recently generated a *Drosophila* model of complex I deficiency using knockdown of the complex I subunit NDUFS1 (ND-75) in *Drosophila* (Granat et al., 2023). Knockdown of ND-75 in this model is highly efficient and results in severe neurological symptoms and very early death, reflecting severe complex I deficiency. Here, we have used an independent RNAi against ND-75 that is much less efficient and causes a weaker knockdown. We find that this weak ND-75 RNAi results in far milder behavioural and cellular phenotypes. Importantly, weak knockdown of ND-75 does not cause changes in endoplasmic reticulum (ER)-mitochondria contacts or activation of ATF4 in neurons. Interestingly, weak ND-75 knockdown in neurons causes significant transcriptional and metabolic changes in the brain but these diverge from strong knockdown of ND-75. The genetic tools that we have characterised provide a powerful system for studying the mechanisms contributing to phenotypic heterogeneity in mitochondrial disease.

## RESULTS

### Manipulation of ND-75 knockdown efficiency using RNAi

**Fig. 1.**
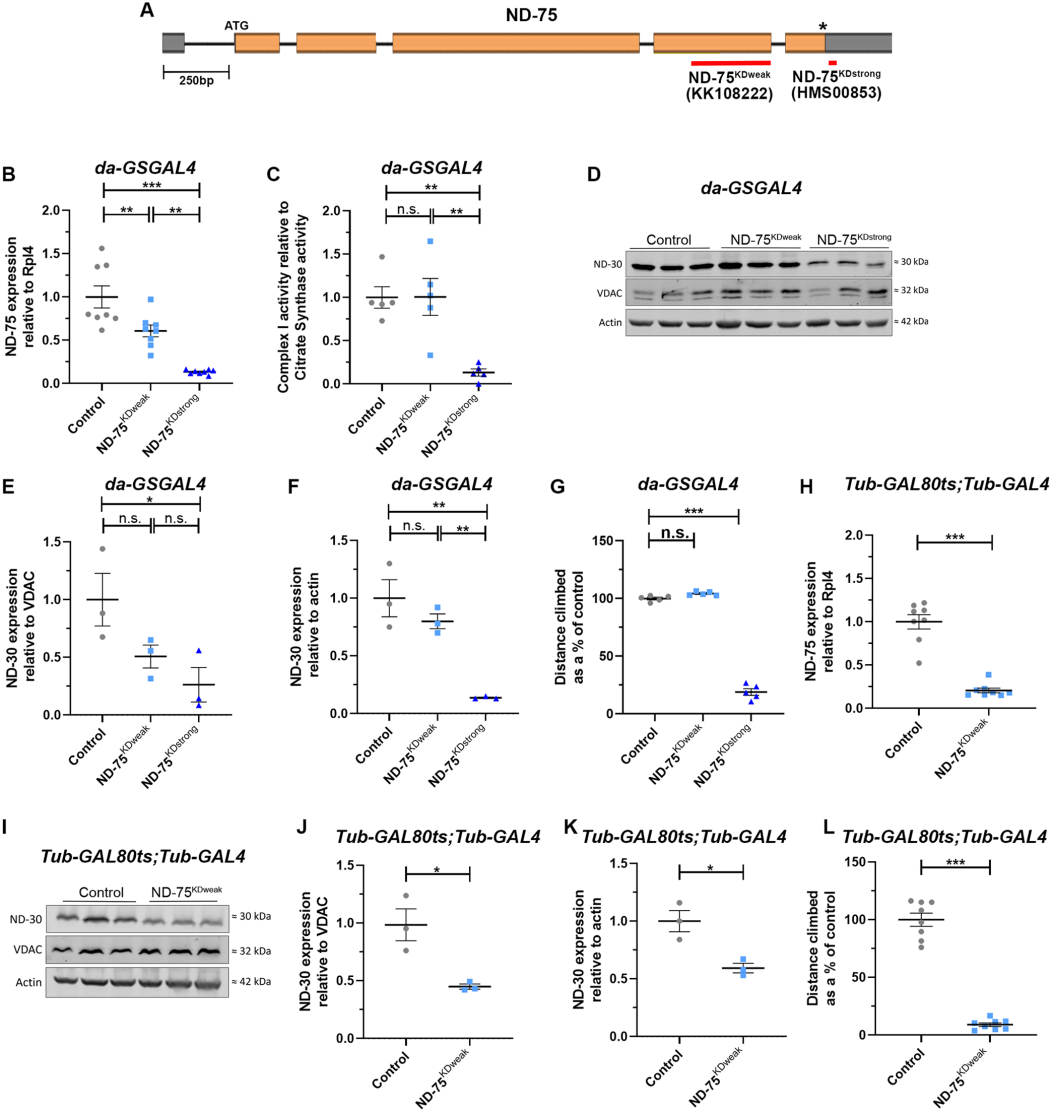
**Analysis of flies with ubiquitous weak and strong knockdown of ND-75.** (A) Diagram showing the *ND-75* genomic region and the areas targeted by ND-75^KDweak^ and ND-75^KDstrong^ RNAis. Coding regions are shown in orange, untranslated regions in grey. Asterisk denotes position of the stop codon. (B) qRT-PCR analysis of ND-75 mRNA levels from flies with ubiquitous expression of ND-75^KDweak^ and ND-75^KDstrong^ in adults using *da-GS-Gal4* for 5 days. *n*=8 biological replicates for all genotypes. (C) Complex I activity in mitochondria isolated from flies with ubiquitous expression of ND-75^KDweak^ and ND-75^KDstrong^ in adults using *da-GS-Gal4* for 5 days. *n*=8 biological replicates for all genotypes. (D) Western blot analysis of ND-30 expression from flies with ubiquitous expression of ND-75^KDweak^ and ND-75^KDstrong^ in adults using *da-GS-Gal4* for 5 days. (E,F) Quantification of ND-30 expression relative to the mitochondrial outer membrane protein VDAC (E) and actin (F), *n*=3 biological replicates for all genotypes. (G) Climbing ability of control flies and flies with ubiquitous expression of ND-75^KDweak^ and ND-75^KDstrong^ in adults using *da-GS-Gal4* for 5 days. *n*=5 flies for all genotypes. (H) qRT-PCR analysis of ND-75 mRNA levels from flies with ubiquitous expression of ND-75^KDweak^ throughout development using *Tub-GAL80^ts^;Tub-GAL4* at 25°C, 2-day-old male flies were used. Control *n*=8, ND-75^KDweak^ *n*=8 flies. (I) Western blot analysis of ND-30 expression from flies with ubiquitous expression of ND-75^KDweak^ throughout development using *Tub-GAL80^ts^;Tub-GAL4* at 25°C, 2-day-old male and female flies were used. (J,K) Quantification of ND-30 expression relative to the mitochondrial outer membrane protein VDAC (J) and actin (K). Control *n*=3, ND-75^KDweak^ *n*=3 biological replicates. (L) Climbing ability of control flies and flies with ubiquitous expression of ND-75^KDweak^ throughout development using *Tub-GAL80^ts^;Tub-GAL4* at 25°C, 2-day-old male flies were used. Control *n*=8, ND-75^KDweak^ *n*=8 flies. Data are represented as mean±s.e.m. and were analysed using a two-tailed students *t*-test or one-way ANOVA with Tukey's *post-hoc* test. n.s not significant, *P<0.5, **P<0.01, ***P<0.001.

Mutations in *NDUFS1* cause complex I deficiency and Leigh syndrome (Koene et al., 2012; Lake et al., 2016). NDUFS1 lies within the N module of complex I and is an [Fe-S] cluster containing core subunit involved in electron transfer (Wirth et al., 2016). To model the phenotypic heterogeneity of complex I deficiency we used two independent non-overlapping ND-75 RNA interference (RNAi) lines with different knockdown efficiencies, ND-75^KK108222^ and ND-75^HMS00853^, hereafter referred to as ND-75^KDweak^ and ND-75^KDstrong^ respectively (Fig. 1A).

To compare the effects of ubiquitous ND-75 knockdown using these two RNAi lines in adult *Drosophila* we expressed ND-75^KDweak^ and ND-75^KDstrong^ using *Tub-Gal4*, however this resulted in embryonic lethality. To circumvent this, we used two strategies: (1) the GeneSwitch system (Roman et al., 2001), using *da-GSGAL4* to restrict ubiquitous ND-75 knockdown to adult flies; (2) ubiquitous expression during development using *Tub-GAL4* together with a temperature-sensitive repressor of Gal4, *Tub-GAL80^ts^,* 25°C [Gal80^ts^ is inactive at 30°C and partially active at 25°C (McGuire et al., 2003)].

Ubiquitous expression of ND-75^KDweak^ and ND-75^KDstrong^ in adult flies with *da-GSGAL4* for 5 days caused a 40% and 88% reduction in ND-75 mRNA levels respectively (Fig. 1B). Analysis of rotenone-sensitive NADH oxidation in mitochondria from adult flies expressing ND-75^KDweak^ with *da-GSGAL4* showed they had similar complex I activity to controls, while flies expressing ND-75^KDstrong^ had an 87% reduction in complex I activity (Fig. 1C). Loss of individual complex I subunits can result in collapse of the whole complex (Ugalde et al., 2004). To test this possibility, we analysed the level of ND-30 expression, the orthologue of mammalian NDUFS4 and a component of Q module of complex I (Garcia et al., 2017). ND-30 expression was unaffected in flies expressing ND-75^KDweak^ with *da-GSGAL4* but ND-30 levels were significantly reduced in flies expressing ND-75^KDstrong^ (Fig. 1D-F). Consistent with these data, ubiquitous expression of ND-75^KDstrong^ but not ND-75^KDweak^ in adult flies using *da-GSGAL4* caused a significant reduction in climbing ability (Fig. 1G).

ND-75^KDstrong^ expression with *Tub-GAL80^ts^;Tub-GAL4* at 25°C caused developmental lethality. Using *Tub-GAL80^ts^;Tub-GAL4* at 25°C to express ND-75^KDweak^ resulted in a high degree of pupal lethality and a small number of viable adult escaper flies. These escaper flies had a 79% reduction in ND-75 expression and, although insufficient in number to isolate mitochondria and analyse complex I activity, showed a significant decrease in ND-30 levels and severely reduced climbing ability (Fig. 1H-L), indicating loss of complex I activity.

Taken together, use of these two ubiquitous expression methods show that loss of complex I activity, the associated loss of ND-30 expression and reduced climbing ability requires at least a 40% reduction in ND-75 gene expression. Moreover, these data demonstrate that both ND-75 RNAi lines are capable of efficient ND-75 knockdown, but ND-75^KDstrong^ is more potent than ND-75^KDweak^.

### Knockdown of ND-75 in neurons models the phenotypic heterogeneity of complex I deficiency

**Fig. 2.**
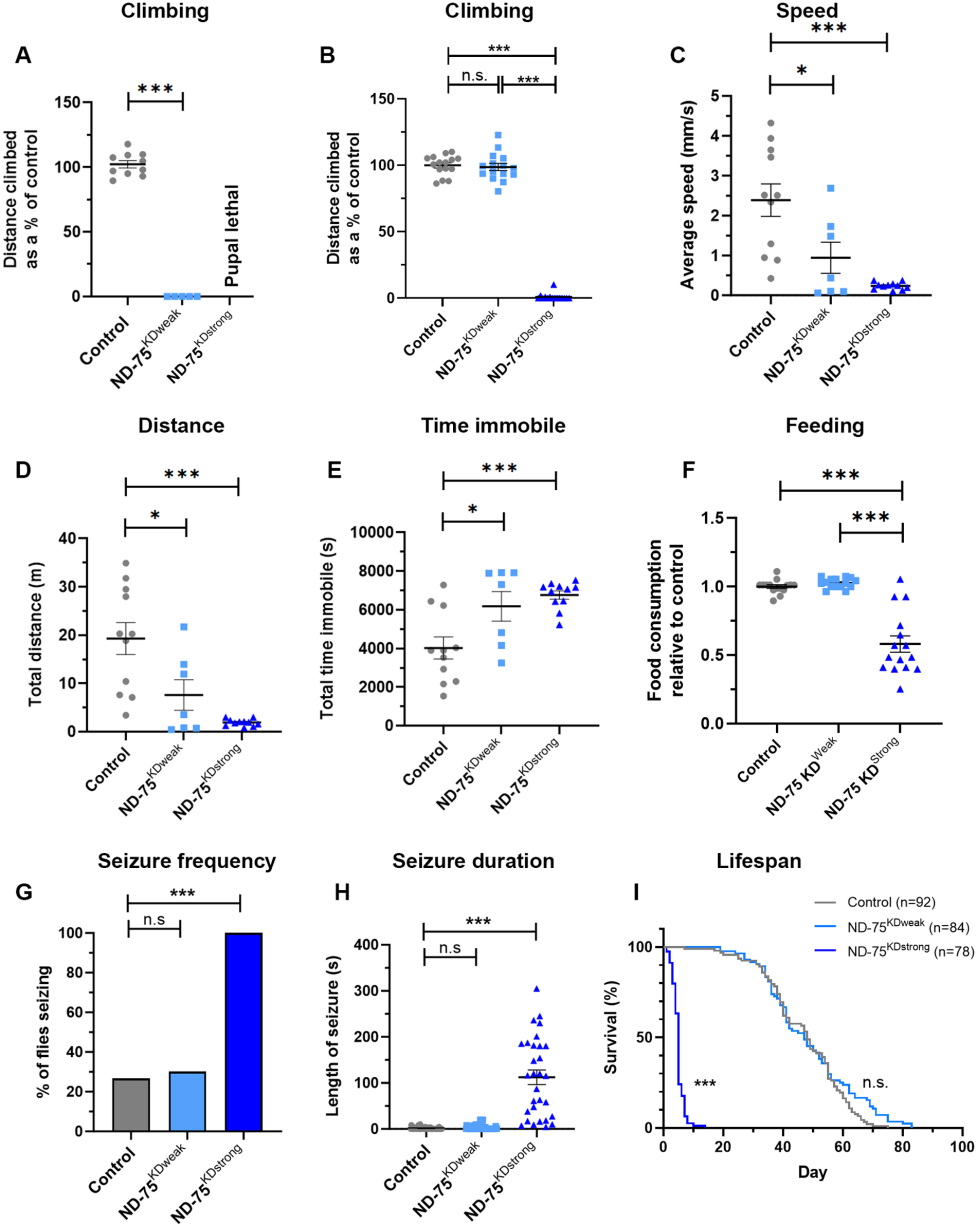
**Pan neuronal weak and strong knockdown of ND-75 models the phenotypic heterogeneity in mitochondrial disease.** (A) Pan-neuronal expression using *nSyb-Gal4* results in flies that are completely unable to climb with ND-75^KDweak^ and lethality with ND-75^KDstrong^. Control *n*=10, ND-75^KDweak^ *n*=5 flies. (B) Pan-neuronal expression using *Gal80^ts^;nSyb-Gal4* at 25°C results in flies that climb normally with ND-75^KDweak^ but are unable to climb with ND-75^KDstrong^. *n*=15 flies for all genotypes. (C-E) Pan-neuronal expression using *Gal80^ts^;nSyb-Gal4* at 25°C causes mild locomotor phenotypes with ND-75^KDweak^ and strong locomotor phenotypes with ND-75^KDstrong^. Control *n*=11, ND-75^KDweak^ *n*=7, ND-75^KDstrong^ *n*=11 flies. (F) Pan-neuronal expression using *Gal80^ts^;nSyb-Gal4* at 25°C causes reduced feeding with ND-75^KDstrong^ but not ND-75^KDweak^, *n*=15 flies for all genotypes. (G,H) Pan-neuronal expression using *Gal80^ts^;nSyb-Gal4* at 25°C causes seizures with ND-75^KDstrong^ but not ND-75^KDweak^. Control *n*=60, ND-75^KDweak^ *n*=63, ND-75^KDstrong^ *n*=30 flies. (I) Pan-neuronal expression using *Gal80^ts^;nSyb-Gal4* at 25°C causes greatly reduced lifespan with ND-75^KDstrong^ but not ND-75^KDweak^. Control *n*=92, ND-75^KDweak^ *n*=84, ND-75^KDstrong^ *n*=78 flies. Males flies were used in A-F and I. Male and female flies were used in G and H. Controls were *nSyb-Gal4* or *Gal80^ts^;nSyb-Gal4* hemizygotes. Data are represented as mean±s.e.m. and were analysed using one-way ANOVA with Tukey's *post-hoc* test, Chi-squared for seizure frequency, or log-rank test for survival curve; n.s. not significant, *P<.0.5, ***P<0.001.

Complex I deficiency primarily affects the nervous system in patients (Koene et al., 2012). To model the phenotypic heterogeneity of complex I deficiency in the nervous system, we induced pan-neuronal expression of ND-75^KDweak^ and ND-75^KDstong^ using *nSyb-GAL4.* ND-75^KDweak^ and ND-75^KDstrong^ expression in neurons using *nSyb-GAL4* caused an inability to climb and pupal lethality respectively (Fig. 2A). In the presence of *Tub-Gal80^ts^* at 25°C, pan neuronal expression of ND-75^KDstrong^ using *nSyb-GAL4* resulted in viable flies that were unable to climb, while expression of ND-75^KDweak^ did not cause a climbing phenotype (Fig. 2B). We next measured open-field behaviour in pan-neuronal ND-75^KD^ flies. The average speed and total distance moved by ND-75^KDstrong^ flies was dramatically reduced, and immobility was increased compared to control flies (Fig. 2C-E). ND-75^KDweak^ flies also had locomotion phenotypes but these were less severe than those observed in ND-75^KDstrong^ flies (Fig. 2C-E).

Complex I deficiency patients have problems with feeding (Koene et al., 2012), and so we measured food intake in our *Drosophila* model. Pan-neuronal ND-75^KDstrong^ flies had a strong reduction in food intake, whereas feeding was unaffected in ND-75^KDweak^ flies (Fig. 2F).

Alongside motor impairments, seizures are frequently reported in complex I deficiency patients (Finsterer and Zarrouk Mahjoub, 2012; Sofou et al., 2014). Using a mechanical stress-induced seizure assay we found that pan-neuronal expression of ND-75^KDstrong^ in neurons caused a dramatic seizure phenotype, with 94% of flies developing seizures, which were significantly longer than controls and, in some cases, lasting 4-5 minutes (Fig. 2G,H). By contrast expression of ND-75^KDweak^ in neurons did not cause a seizure phenotype (Fig. 2G,H).

Complex I deficiency patients typically die within the first few years of life but there is considerable phenotypic heterogeneity (Koene et al., 2012; Bjorkman et al., 2015). Consistent with this, pan-neuronal ND-75^KDstrong^ flies had a dramatically reduced lifespan, with a median survival of 5 days compared to 48 days for controls and a maximum lifespan of 8.6±2.4 days (*n*=8 flies) compared to 67.1±3.6 days for controls (*n*=9 flies). By contrast, the median survival of ND-75^KDweak^ flies was similar to controls (Fig. 2I), while maximum lifespan of ND-75^KDweak^ flies was actually significantly longer than controls (control 67.1±3.6 days, ND-75^KDweak^ 76±4.9 days, *P*=0.0005, *n*=9 flies for both conditions) potentially consistent with studies showing reduced complex I abundance correlating with increased lifespan in mice (Miwa et al., 2014).

Overall, these data show that ND-75^KDweak^ and ND-75^KDstrong^ flies exhibit contrasting behavioural and lifespan phenotypes, mirroring the phenotypic heterogeneity of complex I deficiency patients.

### Strong but not weak ND-75 knockdown disrupts ER-mitochondria contacts in neurons

**Fig. 3.**
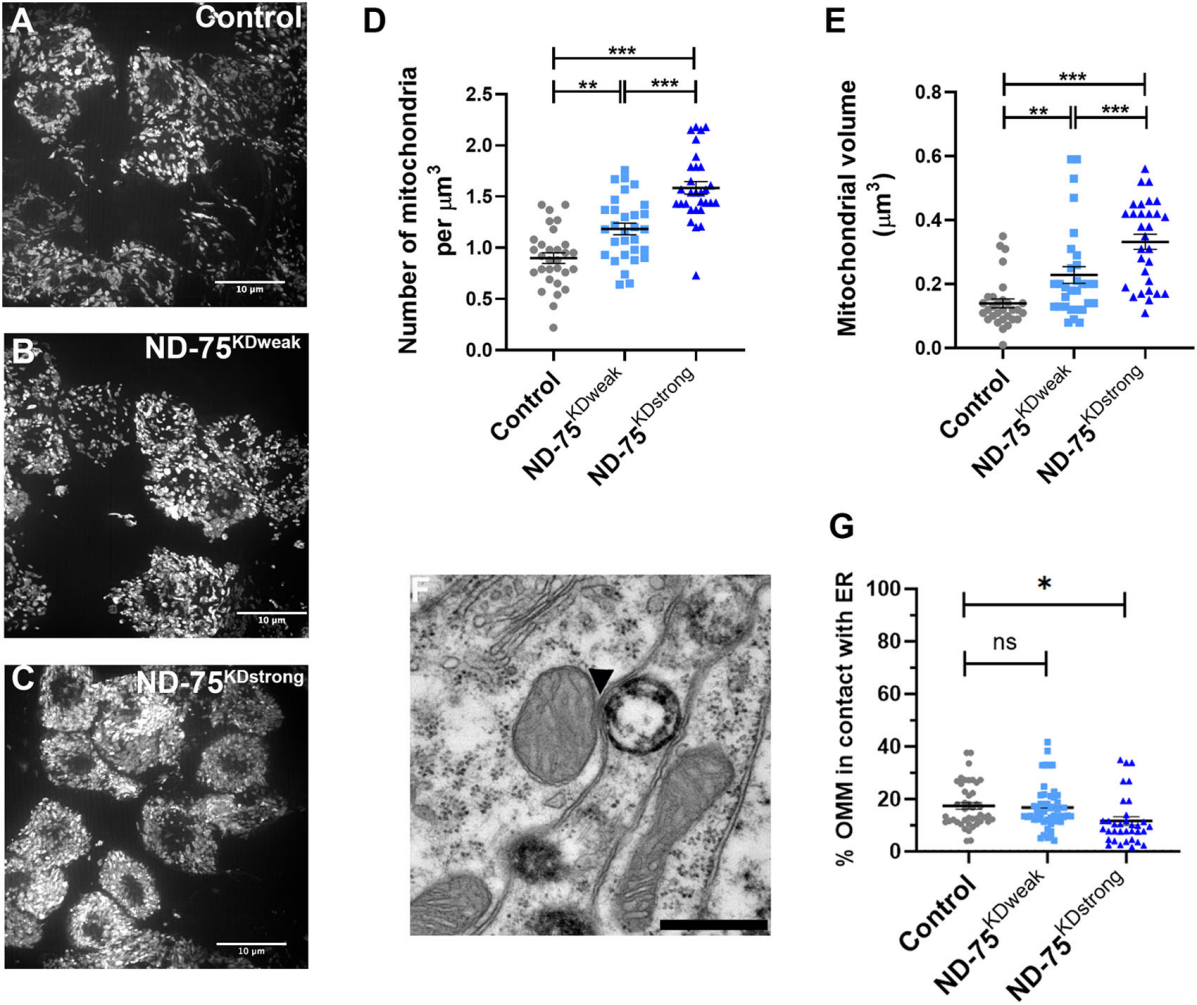
**Weak and strong knockdown of ND-75 have different effects on mitochondrial morphology and ER-mitochondria contacts.** (A-C) Expression of mitochondria-targeted GFP to visualise mitochondria in control (A), ND-75^KDweak^ (B) and ND-75^KDstrong^ (C) larval motor neurons using *OK371-Gal4*. Images were taken using iSIM. Scale bars: 10µm. (D,E) Quantification of mitochondrial number and volume in larval motor neurons with ND-75 knockdown using *OK371-Gal4*. Control *n*=30, ND-75^KDweak^ *n*=30, ND-75^KDstrong^ *n*=30 ROIs. (F) Example transmission electron microscopy image of mitochondria in the adult brain. Arrowhead indicates ER-mitochondria contact. Scale bar: 500 nm (G) Quantification of ER-mitochondria contacts in adult brain from control or with pan-neuronal ND-75 knockdown (using *Gal80^ts^;nSyb-Gal4*). Control *n*=44, ND-75^KDweak^ *n*=49, ND-75^KDstrong^ *n*=34 mitochondria. Controls were *OK371-Gal4* or *Gal80^ts^;nSyb-Gal4* hemizygotes. Data are represented as mean±s.e.m. and were analysed using one-way ANOVA with Tukey's *post-hoc* test. n.s not significant, *P<.0.5, **P<0.01, ***P<0.001.

Mitochondrial dysfunction is associated with perturbed mitochondrial morphology both in model systems and patients (Koopman et al., 2005; Ekstrand et al., 2007; Koopman et al., 2007). We therefore used super resolution imaging of mitochondrially-targeted GFP to analyse mitochondrial morphology. Expression of ND-75^KDstrong^ in larval motor neurons using *OK371-Gal4* severely perturbed mitochondrial morphology, causing dramatically increased mitochondrial number and volume (Fig. 3A-E). Expression of ND-75^KDweak^ caused a much less dramatic but still significant increase in mitochondrial number and volume (Fig. 3A-E).

Mitochondria and the ER are frequently apposed and connected through ER-mitochondria contacts, which can be observed at the ultrastructural level (Fig. 3F) (Stoica et al., 2014). We therefore used transmission electron microscopy to visualise and quantify ER-mitochondria contacts in the adult brain. Pan-neuronal ND-75^KDstrong^ expression using *Tub-Gal80^ts^;nSyb-Gal4* caused a decrease in the number of ER-mitochondria contacts, but ER-mitochondria contacts in pan-neuronal ND-75^KDweak^ flies were similar to control (Fig. 3H). These data suggest that exceeding a threshold of complex I inactivation is required to perturb ER-mitochondria contacts in neurons.

### The ER UPR is activated by strong but not weak ND-75 knockdown in neurons

**Fig. 4.**
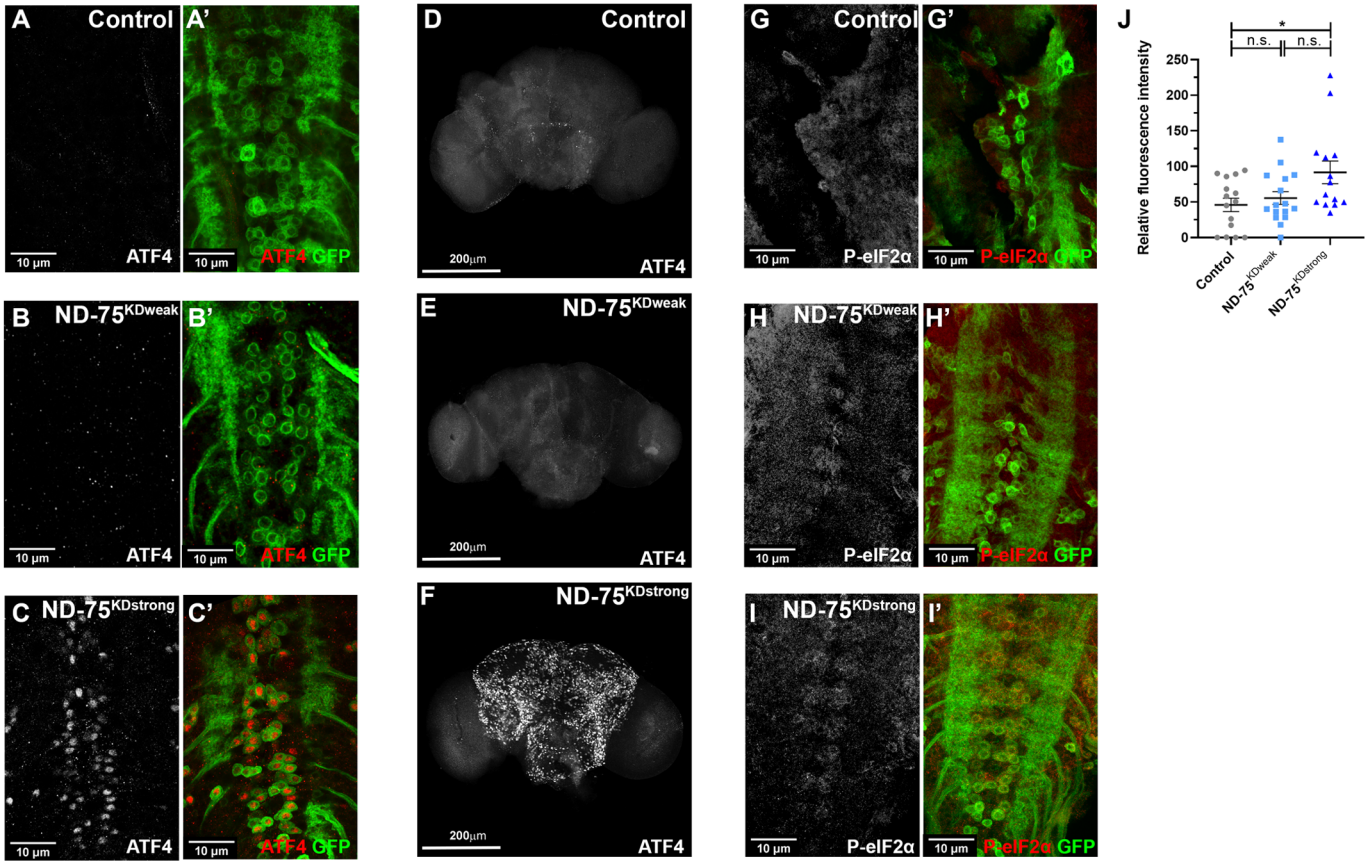
**Strong but not weak ND-75 knockdown activates the ER UPR.** (A-C) ATF4 (red) expression in control (A), ND-75^KDweak^ (B) and ND-75^KDstrong^ (C) larval motor neurons using *OK371-Gal4*. CD8-GFP (green) expression labels motor neurons. (D-F) ATF4 expression in control (D), ND-75^KDweak^ (E) and ND-75^KDstrong^ (F) adult brain tissue using *Tub-Gal80^ts^;nSyb-Gal4*; 1-day-old flies were used. (G-I) Phospho-eIF2α (P- eIF2α, red) expression in control (G), ND-75^KDweak^ (H) and ND-75^KDstrong^ (I) larval motor neurons using *OK371-Gal4*. CD8-GFP (green) expression labels motor neurons. (J) Quantification of Phospho-eIF2α expression. Control *n*=15, ND-75^KDweak^ *n*=16, ND-75^KDstrong^ *n*=14 larval CNS. Data were analysed using ANOVA with Tukey's *post-hoc* test. Controls were *OK371-Gal4* or *Tub-Gal80^ts^;nSyb-Gal4* hemizygotes. Data are represented as mean±s.e.m. n.s., not significant, *P<0.05.

Disruption of ER homeostasis leads to ER stress and activation of the unfolded protein response (UPR) (Oslowski and Urano, 2011). The UPR has been consistently shown to be activated by mitochondrial dysfunction (Hunt et al., 2019; Granat et al., 2020). To investigate whether ND-75 knockdown triggers UPR activation in neurons, we used activating transcription factor 4 (ATF4) expression as a readout of UPR activation. Expression of ND-75^KDstrong^ but not ND-75^KDweak^ using *OK371-Gal4* in larval neurons or *Tub-Gal80^ts^;nSyb-Gal4* in the adult brain caused strong activation of ATF4 expression (Fig. 4A-F). Triggering of the UPR activates protein kinase R-like endoplasmic reticulum kinase (PERK), resulting in ATF4 upregulation through the phosphorylation of the translation initiation factor eIF2α (Hetz and Mollereau, 2014). Consistent with this, ND-75^KDstrong^ but not ND-75^KDweak^ expression increased eIF2α levels in neurons (Fig. 4G-J). Therefore, efficient knockdown of ND-75 activates the UPR and ATF4 in neurons.

### Weak and strong ND-75 knockdown have different effects on transcription and metabolism in the brain

**Fig. 5.**
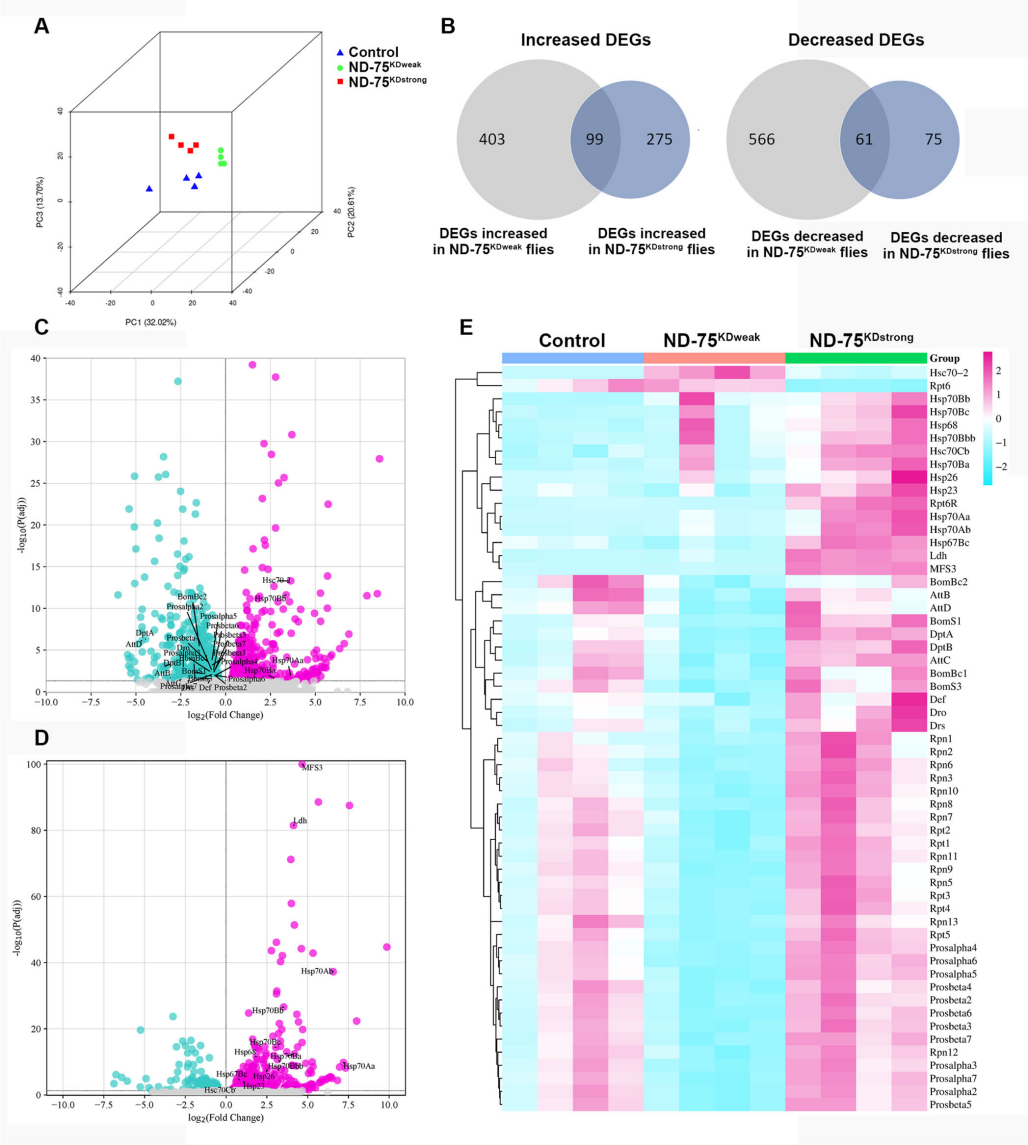
**Weak and strong ND-75 knockdown cause overlapping but distinct transcriptome changes.** (A) PCA plot of transcriptome analysis from head tissue of control, and flies expressing ND-75^KDweak^ and ND-75^KDstrong^ in neurons using *Tub-Gal80^ts^*; *nSyb-Gal4*. (B) Numbers of DEGs increased and decreased in ND-75^KDweak^ and ND-75^KDstrong^ flies and their overlap. (C,D) Volcano plots of DEGs in ND-75^KDweak^ (C) and ND-75^KDstrong^ (D) flies. (E) Heat map showing relative expression of representative genes in ND-75^KDweak^ and ND-75^KDstrong^ flies; 2-day-old male and female flies were used. Controls were *Tub-Gal80^ts^;nSyb-Gal4* hemizygotes.

The activation of ATF4 indicated that strong ND-75 knockdown would result in changes to the transcriptome in neurons. In order to understand the differences in the transcriptional response to pan-neuronal weak and strong ND-75 knockdown using *Tub-Gal80^ts^;nSyb-Gal4*, we performed RNA sequencing of fly heads. Principle component analysis (PCA) showed the two ND-75 knockdown groups were well separated from each other and the control (Fig. 5A). In total there were 1127 differentially expressed genes (DEGs) in ND-75^KDweak^ flies compared to control, of which 501 were up- and 626 genes were downregulated (Fig. 5B,C; Tables S1, S2). Surprisingly, ND-75st^rong^ flies showed a lower number of 508 DEGs (Fig. 5B,D; Tables S3, S4). Of these, 373 were upregulated, while 135 were downregulated (Fig. 5B,D; Tables S3, S4). Although there were fewer DEGs, the average fold change of the upregulated DEGs in ND-75^KDstrong^ flies (FC=8.41) was significantly higher than in ND-75^KDweak^ flies (FC=5.2, *P*=1.56×10^−5^), indicating that strong ND-75 knockdown elicits a more pronounced increase in DEG expression. Strikingly, the first and fourth highest significantly increased genes ranked by adjusted *P* value in ND-75^KDstrong^ flies were *MFS3* and *Ldh*, encoding the glucose/trehalose transporter Major Facilitator Superfamily Transporter 3 and lactate dehydrogenase, respectively (Fig. 5D; Table S3). By contrast, neither *MFS4* nor *Ldh* were differentially expressed in ND-75^KDweak^ flies. Increased *MFS3* and *Ldh* expression indicate a pronounced shift to glycolytic metabolism as a result of strong CI deficiency and activation of ATF4, which regulates *Ldh* expression in *Drosophila* (Lee et al., 2015; Hunt et al., 2019).

Gene ontology (GO) analysis showed ND-75^KDweak^ upregulated DEGs were significantly enriched for genes involved in protein misfolding, oxidoreductase activities, heme/iron binding and lipid catabolic processes, while downregulated DEGs were enriched for proteasome activity and immune response genes (Fig. 5C,E; Fig. S1A, B). GO analysis of upregulated DEGs in ND-75^KDstrong^ highlighted the unfolded protein response, oxidoreductase activities, lipid catabolism and heme binding (Fig. S1C), while no GO categories were significantly downregulated in ND-75^KDstrong^ DEGs.

The molecular function ‘misfolded protein binding’ was enriched in upregulated DEGs in both conditions, however, although the total number of DEGs in ND-75^KDweak^ flies was more than double ND-75^KDstrong^ flies, expression of 11 heatshock genes (*Hsp23, Hsp26, Hsp67Bc, Hsp68, Hsp70Aa, Hsp70Ab, Hsp70Ba, Hsp70Bb, Hsp70Bbb, Hsp70Bc*) were significantly increased in ND-75^KDstrong^ flies while only four (*Hsc70-2, Hsp70Aa, Hsp70Ba, Hsp70Bb*) were increased in ND-75^KDweak^ flies (Fig. 5D,E; Tables S1, S3). These data suggest that strong CI deficiency elicits a greater chaperone response than weak CI deficiency in the brain.

Subunits of the 26S proteasome (e.g. *Prosalpha4, Prosalpha5, Prosbeta7*) were strongly enriched in the ND-75^KDweak^ downregulated DEGs, whereas no 26S proteasome subunits were downregulated in ND-75^KDstrong^ flies (Fig. 5C-E; Tables S2, S4). The expression of many different antimicrobial peptides (e.g. *AttB*, *AttD*, *DptA*) were downregulated in ND-75^KDweak^ flies, whereas only one (*Drsl4*) was downregulated in ND-75^KDstrong^ flies (Fig. 5C-E; tables S2, S4). Therefore, weak CI deficiency specifically represses proteosome activity and immune response genes in the brain.

**Fig. 6.**
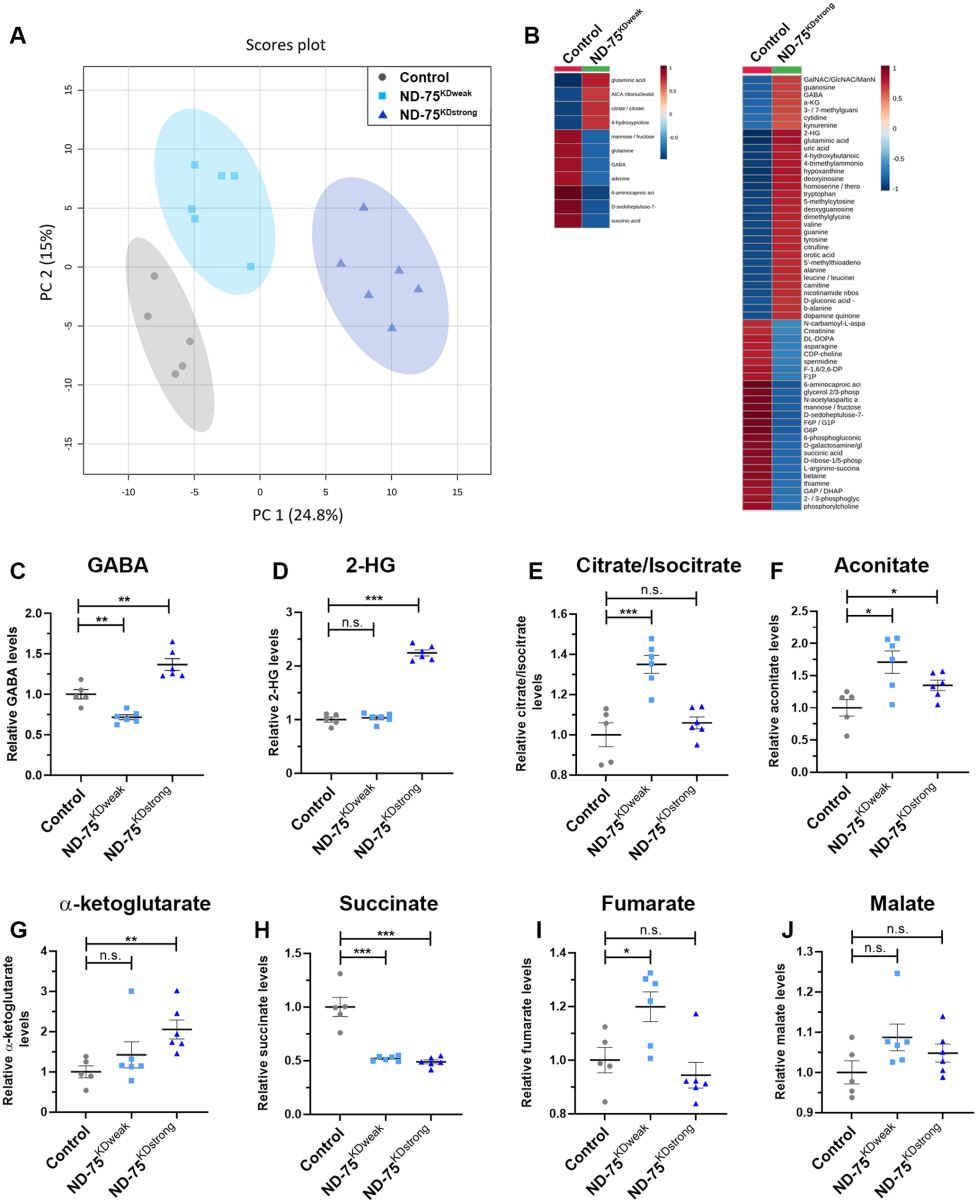
**Metabolic reprogramming in neurons is dependent on the level of ND-75 knockdown.** (A) PCA plot of metabolomic analysis from head tissue of control, and flies expressing ND-75^KDweak^ and ND-75^KDstrong^ in neurons using *Tub-Gal80^ts^*; *nSyb-Gal4*. (B) Metabolites significantly misregulated in ND-75^KDweak^ and ND-75^KDstrong^ flies. Scale represents the z score. (C,D) Levels of GABA (C) and 2-HG (D) in ND-75^KDweak^ and ND-75^KDstrong^ flies. (E-J) Levels of TCA cycle metabolites in ND-75^KDweak^ and ND-75^KDstrong^ flies; 2-day-old male and female flies were used. Control *n*=5, ND-75^KDweak^ *n*=6, ND-75^KDstrong^ *n*=6 biological replicates. Data were analysed using ANOVA with Tukey's *post-hoc* test. Controls were *Tub-Gal80^ts^*; *nSyb-Gal4* hemizygotes. Data are represented as mean±s.e.m; n.s. not significant, *P<0.05, **P<0.01, ***P<0.001.

Complex I deficiency causes profound alterations in brain metabolism (McElroy et al., 2020; van de Wal et al., 2022; Granat et al., 2023). In keeping with this, our transcriptomic analysis showed ND-75 knockdown results in altered expression of metabolic genes such as *Ldh*. To understand how weak and strong complex I deficiency in *Drosophila* neurons affects metabolism, we performed untargeted metabolomics of head tissue from flies expressing ND-75^KDweak^ and ND-75^KDstrong^ in neurons using *Gal80^ts^;nSyb-Gal4.* PCA revealed that knockdown of ND-75 using either ND-75^KDweak^ or ND-75^KDstrong^ produced metabolic profiles that were distinct from control (Fig. 6A). The profiles were also distinct from each other, with ND-75^KDweak^ bearing more similarity to controls than ND-75^KDstrong^ (Fig. 6A).

Of the 195 metabolites identified (Table S5), expression of ND-75^KDweak^ led to a significant upregulation in the levels of four metabolites, and a significant downregulation in the levels of seven metabolites compared to controls (Fig. 6B, Table S6). Expression of ND-75^KDstrong^ led to a significant up-regulation in the levels of 32 metabolites, and a significant downregulation in the levels of 26 metabolites compared to controls (Fig. 6B, Table S7). Six of the 11 metabolites mis-regulated in ND-75^KDweak^ flies were also mis-regulated in ND-75^KDstrong^ flies. Interestingly, five of these were similarly increased or decreased in both conditions (6-aminocaproic acid, D-sedoheptulose-7-phosphate, glutaminic acid, succinic acid and mannose/fructose), whereas gamma-aminobutyric acid (GABA) levels were decreased in ND-75^KDweak^ flies but increased in ND-75^KDstrong^ flies (Fig. 6C; Fig. S2A,B; Tables S6, S7). As the major inhibitory neurotransmitter, GABA may contribute to the behavioural defects caused by strong ND-75 knockdown.

We performed metabolite set enrichment analysis (MSEA) to identify biological processes or molecular pathways that were significantly dysregulated (Xia and Wishart, 2010). ND-75^KDweak^ expression led to significant dysregulation in 22 of 54 (41%) human KEGG pathways (Fig. S2C). In comparison, ND-75^KDstrong^ expression caused significant dysregulation in 44 of 54 KEGG pathways examined (81%) (Fig. S2D). Comparison of the weak and strong ND-75 knockdown MSEA highlighted several processes that were dysregulated specifically in ND-75^KDstrong^ flies including the pentose phosphate pathway, pantothenate and CoA biosynthesis, beta alanine metabolism, lysine degradation and galactose metabolism (Fig. S2C,D). Moreover, 2-hydroxyglutarate (2-HG) levels were strongly increased in ND-75^KDstrong^ but not ND-75^KDweak^ flies (Fig. 6D). 2-HG is synthesised from the TCA cycle intermediate α-ketoglutarate and we and others have shown 2-HG levels are increased as a consequence of mitochondrial dysfunction and contribute to neuronal dysfunction (Burr et al., 2016; Hunt et al., 2019).

MSEA revealed that the TCA cycle was dysregulated in both ND-75^KDweak^ and ND-75^KDstrong^ flies (Fig. S2C,D). Mitochondrial complex I is functionally coupled to the TCA cycle. NADH oxidation by FMN within the matrix arm of complex I replenishes NAD+, a co-factor required for the generation of α-ketoglutarate from isocitrate, succinyl-CoA from α-ketoglutarate, and oxaloacetate from malate (Fig. S2E). Expression of ND-75^KDweak^ led to a significant rise in citrate/isocitrate levels (Fig. 6E), whereas expression of ND-75^KDstrong^ had no significant effect (Fig. 6E). In contrast, both the ND-75^KDweak^ and ND-75^KDstrong^ induced a significant increase in aconitate, an intermediate in the conversion of citrate to isocitrate (Fig. 6F). Consistent with the changes in 2-HG levels, expression of ND-75^KDstrong^ but not ND-75^KDweak^ caused a significant elevation in α-ketoglutarate levels (Fig. 6G), and both produced a significant, comparable reduction in succinate (succinic acid) levels (Fig. 6H). Expression of ND-75^KDweak^, but not ND-75^KDstrong^ led to a significant increase in fumarate levels (Fig. 6I), whereas neither had any significant effect on malate levels (Fig. 6J). Succinyl-CoA and oxaloacetate, the remaining TCA cycle intermediates, were not detected. Taken together, these data demonstrate that neuronal-specific knockdown of ND-75 leads to significant metabolic abnormalities in a range of metabolic pathways, including the TCA cycle, and that the severity of metabolic dysfunction is dependent on the extent of ND-75 knockdown.

## DISCUSSION

Primary mitochondrial diseases have a common cause but are highly heterogeneous. We have modelled this heterogeneity using a knockdown approach in *Drosophila*. Using two independent ND-75 RNAi lines, our data show that the behavioural phenotypes and mitochondrial morphology defects caused by complex I deficiency in neurons correlate with the ND-75 knockdown efficiency. However, the loss of ER-mitochondria contacts and activation of the UPR only occur with strong ND-75 knockdown. The transcriptional and metabolic changes caused by weak and strong ND-75 knockdown provide mechanistic insight and highlight specific molecular functions and metabolic pathways that differentiate the heterogeneity in complex I deficiency. Thus, our *Drosophila* model has good face validity for complex I deficiency and provides new insight into the cellular and molecular mechanisms associated with phenotypic heterogeneity.

Complex I deficiency typically manifests with neonatal-onset lactic acidosis or encephalomyopathies, Leigh syndrome, leukoencephalopathy, hypertrophic cardiomyopathy, exercise intolerance and is often fatal (Fassone and Rahman, 2012). However, heterogeneity has been documented in patients with mutations in several different complex I subunits. Missense mutations in the assembly factor NDUFAF5 cause low complex I activity resulting in classical early onset Leigh syndrome with onset before 6 months and death by 3 years (Gerards et al., 2010). However, several adult patients have been reported with more mild loss of complex I activity and minor neurological involvement (Gerards et al., 2010; Simon et al., 2019). Moreover, paediatric patients with mutations in the complex I accessory subunit NDUFA12 exhibited a range in the onset of motor symptoms (Torraco et al., 2021). Patients with NDUFS1 mutations most frequently manifest with severe neonatal rapidly progressive leukoencephalopathy that is fatal. However, a 7-year-old patient with a missense mutation in NDUFS1 had developmental delay and early motor symptoms associated with infections that gradually improved to the point where he was in generally good health (Kashani et al., 2014). This patient's fibroblasts showed complex I activity was reduced to 20% of control cells. Björkman et al., described three patients with NDUFS1 mutations, two with severe symptoms who died within 6 weeks of birth and a third 3.5-year-old with much milder symptoms, including hypertonia, who had normal language development and crawling (Bjorkman et al., 2015). Pyruvate+malate and NADH ferricyanide reductase activities in isolated skeletal muscle mitochondria were outside the normal range but more mildly affected in this patient than the severe cases (Bjorkman et al., 2015). Although limited in number, these clinical studies demonstrate that the mild behavioural phenotypes in ND-75^KDweak^ flies reflect the symptoms caused by rare NDUFS1 mutations that likely cause a less severe loss of complex I activity.

The two ND-75 RNAi lines used both target non-overlapping regions towards the end of the gene (Fig. 1A). The difference in efficiency between the two RNAis can likely be explained by their properties; ND-75^KDstrong^ encodes a short hairpin RNA (shRNA), whereas ND-75^KDweak^, encodes a long hairpin RNA (lhRNA) (Fig. 1A). Previous comparisons of shRNAs and lhRNAs targeting same gene have revealed that shRNAs consistently produce stronger phenotypes than lhRNAs (Ni et al., 2011; Bartoletti et al., 2017), strongly suggesting that shRNAs, which mimic endogenous microRNAs, are more efficient at reducing mRNA levels. Consistent with this, ubiquitous knockdown (using *Da-Gal4*) of ND-75 with an independent lhRNA produced viable flies with an approximately 50% reduction in lifespan (Hegde et al., 2014). It will be interesting in future to employ the RNAi lines we have characterised to model the effects of complex I deficiency and phenotypic heterogeneity in other tissues, such as muscle, affected in mitochondrial disease patients.

ND-75^KDstrong^ flies had loss of climbing ability, severe loss of locomotor function, seizures and greatly reduced lifespan. By contrast, the only behavioural defect in ND-75^KDweak^ flies was a mild reduction in locomotor function. However, several cellular and molecular changes were evident in ND-75^KDweak^ flies, including increased mitochondrial number and volume in neurons, 1127 DEGs and altered levels of 11 metabolites in the brain. Significantly, ND-75^KDweak^ expression did not activate ATF4, suggesting that the severe behavioural phenotypes caused by strong ND-75 knockdown are associated with ATF4 expression in neurons. Consistent with this, expression of *Ldh*, which is an ATF4 target in *Drosophila* (Lee et al., 2015; Hunt et al., 2019), is strongly increased in ND-75^KDstrong^ but not in ND-75^KDweak^ flies. Moreover, we previously showed in an independent *Drosophila* mitochondrial dysfunction model that activation of ATF4 in neurons causes increased 2-HG levels in the brain (Hunt et al., 2019). 2-HG is also increased in cells from patients with a form of Leigh syndrome (Burr et al., 2016). Interestingly, metabolomic analysis of children with mutations in respiratory chain complex I, complex III or multiple complex deficiencies showed they had significantly increased 2-HG levels in urine (Reinecke et al., 2012). Thus, 2-HG may have potential as a prognostic biomarker for severe mitochondrial disease involving activation of ATF4.

The transcriptomic and metabolomic data also show that large scale transcriptional and limited metabolic changes in the brain of ND-75^KDweak^ flies occur in the absence of ATF4 activation. These transcriptional and metabolic changes indicate distinct (e.g. proteosome activity and immune response genes) responses to mild complex I deficiency that do not involve UPR activation. It will be interesting in future to investigate the signalling pathways and transcription factors responsible.

A limitation of our study is that we do not know the knockdown efficiency and extent of complex I deficiency in ND-75^KDweak^ and ND-75^KDstrong^ neurons. Analysis of ND-75 expression in neurons would require isolation of pure neuronal populations and this is not practical for the quantity of isolated mitochondria required to analyse complex I activity. Given loss of complex I activity in whole flies requires at least a 40% reduction in ND-75 gene expression it is possible that the behavioural phenotypes in ND-75^KDweak^ flies are caused by reduced ND-75 expression independent of its role in OXPHOS. This may involve structural alterations to complex I or other effects that will be interesting in future to explore. Another limitation is that we did not use a control RNAi line, which could potentially undermine some of the transcriptional and metabolic findings. However, as many of the changes we observe in ND-75 knockdown flies, such as increased glycolytic metabolism and stress response genes, increased 2-HG levels and altered TCA cycle metabolites, are consistent with other independent studies of mitochondrial dysfunction/disease models our transcriptional and metabolic findings are likely to be robust.

Overall, our study illustrates how using RNAis with different efficiencies to knockdown the same OXPHOS subunit in *Drosophila* provides a powerful means of modelling mitochondrial disease phenotypic heterogeneity. It will be interesting to compare these findings with future studies in complex I deficiency patients and of patient-derived cells to determine the predictive power of our *Drosophila* model.

## MATERIALS AND METHODS

### Fly strains and growth conditions

Flies were maintained on standard food [per litre: 6.4 g Agar (ThermoFisher Scientific), 64 g glucose (Sigma-Aldrich), 16 g ground yellow corn and 80 g Brewer's yeast (MP Biomed Europe), 3 ml propionic acid (ThermoFisher Scientific), 1.8 g methyl 4-hydroxybenzoate (Sigma-Aldrich), 16 ml ethanol (Sigma-Aldrich)] at 25°C in a 12 h light:dark cycle unless stated otherwise. Genotypes for all experiments are described in Table S8. ND-75^KDweak^ (ND-75^KK108222^) was from the Vienna *Drosophila* Resource Center (Dietzl et al., 2007), ND-75^KDstrong^ (ND-75^HMS00853^) was from the TRiP collection (Perkins et al., 2015) and obtained from the Bloomington Stock Center. *Daughterless-GeneSwitch-GAL4* and *Tub-Gal80^ts^; nSyb-Gal4* expression were performed as in (Granat et al., 2023).

### Behavioural analysis

For behavioural assays with ND-75^KDstrong^ flies, vials were placed on their sides during eclosion to prevent flies from becoming stuck in the food.

Climbing assays were performed using male flies as in (Hunt et al., 2019). Briefly, individual flies were aspirated from a vial into a 10 ml serological pipette (Falcon). Flies were relocated to the base of the pipette by tapping against the bench. The height obtained in three, continuous, 10 s climbs was measured for each fly, and the mean calculated. Wandering (i.e. non-vertical) or discontinuous climbs were excluded.

Open-field locomotor activity, seizure, lifespan and CApillary FEeder assay (CAFE) analysis was performed as in (Granat et al., 2023). Locomotor assays were performed the following morning, 1–4 h after the start of the 12 h light cycle. Flies were briefly anaesthetised on ice and placed into individual open-field arenas 35 mm in diameter and 1.8 mm in height with vibrating motors attached (Kottler et al., 2019). Flies were left to acclimatise to the arenas for 15 min prior to the start of video recording. Flies were video recorded for a total of 2 h 15 min: after an initial rest period of 30 min, flies were subjected to a mechanical stimulus (five vibrations, 0.2 s long, 0.5 s apart) followed by 15 min recovery. This pattern of stimulus and recovery was repeated a further five times before a final rest period of 15 min. Tracking of individual flies and analysis of average speed, total distance and immobility was performed using Anymaze software (Stoelting).

Mechanical stress-induced seizure assays were performed using both male and female flies. Flies of the desired genotype were collected on the day of eclosion and added to fresh vials so that each vial contained a total of 2 flies. The following day, flies were transferred to an empty vial, and vortexed at full speed for 10 s. Immediately after vortexing, flies were observed for seizures. A seizure was defined as a period of paralysis, potentially interspersed with limb twitching, wing movements and abdominal contractions. The length of each seizure was recorded, with the seizure considered to have ended when the fly stood upright.

Lifespan assays were performed using male flies. Male flies of the desired genotype were collected on the day of eclosion and added to fresh vials. Ten flies were initially added to each vial, and flies were incubated on their sides at 25°C for the duration of the assay. Every 2-3 days, flies were flipped into fresh vials and the number of dead flies in each vial were recorded. Dead flies that were carried over into the fresh vials were deducted from the next death count. Any flies that escaped during flipping, or were alive but stuck in the food so could not be flipped, were censored and deducted from the total fly count. Vials were flipped until all residing flies were dead.

To measure food intake, the CAFE assay was performed using 10 male and female flies per container for 24 h as in (Diegelmann et al., 2017).

### qRT-PCR

Four adult flies were used per biological replicate. Samples were first homogenised in 100 µl TRIzol (ThermoFisher Scientific) using a pestle and left to sit at room temperature for 5 min. 20 µl chloroform (Sigma-Aldrich) was then added and mixed by shaking thoroughly. Samples were left at room temperature for a further 3 min and then spun down at 12,000 g for 15 min at 4°C. The upper phase was then transferred into a separate Eppendorf containing 50 µl isopropanol, vortexed, and incubated at −70°C for 20 min. Samples were next spun at 12,000 g for 10 min at 4°C. The resulting supernatant was discarded, and 200 µl 75% ethanol was added to remaining RNA pellets. Samples were then spun at 12,000 g for 5 min at room temperature, the ethanol was removed, and the RNA was resuspended in 32 µl RNAse-free ddH_2_O. RNA concentrations were then measured using a NanoDrop spectrophotometer (ThermoFisher Scientific). Any remaining DNA was removed from the RNA samples using a DNase I amplification grade kit (AMPD1, Sigma-Aldrich) by following manufacturer's protocols. cDNA was then synthesised from 10 µl RNA using the first strand cDNA synthesis kit (K1612, ThermoFisher Scientific), following manufacturers protocols. cDNA was diluted to 10 ng/µl. The mix for each qPCR reaction was as follows: 5 µl qPCRBIO SyGreen qPCR mix Lo-ROX (PCR Biosystems), 0.4 µl forward primer, 0.4 µl reverse primer and 4.2 µl cDNA. Each sample was run in triplicate on a 384-well plate. qPCRs were performed using a QuantStudio 7 Flex real-time PCR system (Applied Biosystems) and the following program: fast, 95°C for 2 min, followed by 40 cycles of 95°C for 5 s and 55°C for 20 s. Melt curves were produced at the end of each run using the following temperature steps: 95°C for 15 s, 60°C for 60 s, 95°C for 15 s.

Data were analysed using QuantStudio Software v.1.3. The 2^-ΔΔCT^ method was used. Technical replicates were considered outliers and excluded if the Ct value was more than 0.3 outside of the range of the other two replicates, or if melt curves produced multiple peaks. The following primers were used:

ND-75 forward: 5′-ACATTAACTACACGGGCAAGC-3′

ND-75 reverse: 5′- CAATCTCGGAGGCGAAAC-3′

Rpl4 forward: 5′-TCCACCTTGAAGAAGGGCTA-3′

Rpl4 reverse: 5′-TTGCGGATCTCCTCAGACTT-3′

### Mitochondrial NADH dehydrogenase (complex I) assay

Flies were snap-frozen in liquid nitrogen and stored at −70°C until required. For each replicate, 30 females and 30 males were used. Mitochondrial complex I and citrate synthase activity were assessed using a published protocol (Spinazzi et al., 2012). For detailed methods on how the complex I assay was performed see (Granat et al., 2023).

### Immunofluorescence and imaging

Images were taken using a Nikon A1R confocal microscope or a Nikon Vt-iSIM super resolution microscope with NIS Elements software. Confocal imaging and quantification was performed as in (Granat et al., 2023). A Vt-iSIM super resolution microscope (Nikon) was used to capture mitoGFP expression as in (Granat et al., 2023). Primary antibodies were rat anti-ATF4 (1:200, Hunt et al., 2019) and rabbit anti-P-eIF2α (1:500, anti-Phospho-eIF2α [Ser51], Cell Signaling Technology 9721). Secondary antibodies were goat anti-rabbit Alexa Fluor 546 (1:1000, Invitrogen A11035) and goat anti-rat Alexa Fluor 555 (1:1000, ThermoFisher Scientific A21434).

### Western blot analysis

For each biological replicate, 10 male and 10 female flies were used. Flies were first homogenised in 40 µl 1× sample buffer using a pestle in a 1.5 ml Eppendorf and spun down at 14,000 ***g*** for 5 min. The 1× sample buffer consisted of 50 mM Tris-HCl pH 6.8, 10% v/v glycerol, 2% w/v SDS and 0.01% w/v bromophenol blue. 100 mM dithiothreitol (DTT) was added and samples were boiled at 95°C for 5 min. Samples were stored at −20°C. SDS-PAGE gels were run at 70 V for 30 min, and then at 110 V for 1–2 h. Protein was then transferred onto an Amersham nitrocellulose membrane (GE Healthcare Life Sciences) at 75 V for 1.5 h (for proteins of interest <100 kDa) or 2.5 h (for proteins of interest >100 kDa). Membranes were next blocked for a minimum of 30 min at room temperature in 5% (w/v) skimmed milk in Tris-buffered saline (pH 7.4, TBS), or for phosphorylated proteins, in 5% (w/v) BSA in TBS. Membranes were then incubated overnight at 4°C with the relevant primary antibody. Primary antibodies were diluted in 5% (w/v) BSA in TBS plus 0.1% TritonX-100 (TBS-T) and 0.2% sodium azide. The following day, membranes were washed three times for 5–10 min with TBS-T and incubated for 1 h at room temperature with secondary antibody. Secondary antibodies were diluted in 2% (w/v) skimmed milk in TBS-T. Membranes were subsequently washed twice in TBS-T and a final time in TBS, before being analysed using an Odyssey CLx near-infrared imaging system (Li-cor). Antibody signals were quantified using the Image Studio software (Li-cor) ‘analysis’ tab. Firstly, rectangles were drawn around the relevant protein band. The background subtraction was then set to ‘median’, with a border width of 1. The intensity of each band was then recorded. Antibodies used were rabbit anti-VDAC (1:1000; ab14374, Abcam), mouse anti-Ndufs3 (1:500; ab14711, Abcam) and rabbit anti-actin (1:5000; 4967, Cell Signalling Technology). Secondary antibodies were IR Dye 680RD goat anti-rabbit IgG (1:10,000; 926-68071, Licor) and IR Dye 800CW goat anti-mouse IgG (1:10,000; 925-32210, Licor).

### RNA sequencing (RNA-Seq) transcriptomic analysis

Twenty snap-frozen fly heads (10 male and 10 female, 2 days old) were used for each replicate and placed into 100 µL of lysis buffer+β-mercaptoethanol from the Absolutely RNA Microprep kit (Agilent Technologies). Each genotype was prepared in quadruplicate. RNA was extracted from using the Absolutely RNA Microprep kit according to the manufacturer's protocol. The samples were sent on dry ice to Novogene Ltd. Sequencing libraries were generated using NEBNext Ultra TM RNA Library Prep Kit for Illumina (NEB, USA) following manufacturer's recommendations. RNA-seq was performed as described previously (Granat et al., 2023). Genes with an adjusted *P* value<0.05 were assigned as differentially expressed. GO enrichment was performed using the DAVID Knowledgebase (https://david.ncifcrf.gov/tools.jsp). Heatmaps and volcano plots were generated using SRPlot (Tang et al., 2023). RNA-Seq data have been deposited in NCBI's Gene Expression Omnibus (GEO) (Edgar et al., 2002) and are accessible through GEO Series accession number GSE248363.

### Metabolomic analysis

Twenty 2–5-day-old adult flies (equal numbers of males and females) were snap frozen on liquid nitrogen in a 15mL Falcon tube and then vortexed for 5 s five times to decapitate. Heads were then quickly separated and stored at −80°C. For a detailed description of the metabolomics method see (Hunt et al., 2019). Data acquisition and analysis were carried out by Xcalibur 4.1 software and Tracefinder 4.1 software, respectively (both ThermoFisher Scientific). The peak area for each detected metabolite was normalized by the total ion count. Metabolomic data were analysed using MetaboAnalyst v.5.0 (Chong et al., 2018).

### Transmission electron microscopy (TEM)

Two-day-old dissected adult fly brain samples were post-fixed with 1% (w/v) osmium tetroxide and 1% potassium ferrocyanide (w/v) in 0.1 M sodium phosphate buffer (pH 7.4) for 1 h before being washed and dehydrated through a graded acetone series. Samples were then infiltrated with increasing concentrations of SPURR epoxy resin/acetone mixture before being placed into 100% resin overnight with rotation. The following day, the samples were infiltrated further before embedding (with the dorsal face orientated toward the sectioning plane) and polymerised at 70°C for 24 h.

Ultrathin sections (50–70 nm) were prepared using a Leica UC7 ultramicrotome (Leica microsystems, Vienna), mounted on grids and contrasted using UranyLess (22,409 Electron Microscopy Sciences, USA) and lead citrate (22410 Electron Microscopy Sciences, USA). Samples were examined on a JEOL JEM 1400 Flash (JEOL, Japan) transmission microscope operated at 80 kV and images were acquired with a JEOL Flash Camera.

Mitochondrial contacts were quantified in ImageJ (version 1.52). Mitochondria with ER contacts were identified by observing a 30 nm or less distance between the mitochondria and ER. For positive contacts, mitochondria circumferences were measured and recorded. The length of the ER with a distance <30 nm was also measured and then divided by the mitochondria circumference to calculate the proportion of the mitochondrial membrane in contact with the ER.

### Statistical analyses

Continuous data are expressed as mean±s.e.m. unless stated otherwise. Non-continuous data are expressed as percentages unless stated otherwise. All data apart from transcriptomic and metabolomic were analysed using Prism 8 (GraphPad). Student's unpaired two-way *t*-tests were used for pairwise comparisons of continuous data. An *F*-test was used to test for unequal variances, and where significant, Welch's correction was applied to the *t*-test. A one-way ANOVA with Tukey's post-hoc test was used for continuous data with multiple comparisons. Chi-squared test and Fisher's tests were used for non-continuous data, and were applied to the raw values rather than percentages. The log-rank test was used for lifespan data. Maximum lifespan was calculated as the average lifespan of the most long-lived 10% of flies for each genotype. For the NADH dehydrogenase activity assay data were normalised to the control and then transformed using log base 2. *P*-values <0.05 were considered significant; *P<0.05, **P<0.01, ***P<0.001.

## Supplementary Material

10.1242/biolopen.060278_sup1 Supplementary information

Table S1. Genes with significantly increased expression in head tissue from flies with pan-neuronal ND-75^KDweak^ knockdown using Tub-Gal80^ts^; nSyb-Gal4. Ranked by adjusted p value.

Table S2. Genes with significantly decreased expression in head tissue from flies with pan-neuronal ND-75^KDweak^ knockdown using Tub-Gal80^ts^; nSyb-Gal4. Ranked by adjusted p value.

Table S3. Genes with significantly increased expression in head tissue from flies with pan-neuronal ND-75^KDstrong^ knockdown using Tub-Gal80^ts^; nSyb-Gal4. Ranked by adjusted p value. MFS3 and Ldh highlighted in red.

Table S4. Genes with significantly decreased expression in head tissue from flies with pan-neuronal ND-75^KDstrong^ knockdown using Tub-Gal80^ts^; nSyb-Gal4. Ranked by adjusted p value.

Table S5. Metabolite levels, normalised to total ion count, from control, pan-neuronal ND-75^KDweak^ or ND-75^KDstrong^ fly head tissue using Tub-Gal80^ts^; nSyb-Gal4. Colour of metabolite ID stands for: black = identity confirmed by standard or MS2, red = cannot separate, orange = identity not confirmed.

Table S6. Metabolites with significantly altered levels in head tissue from flies with pan-neuronal ND-75^KDweak^ knockdown using Tub-Gal80^ts^; nSyb-Gal4.

Table S7. Metabolites with significantly altered levels in head tissue from flies with pan-neuronal ND-75^KDstrong^ knockdown using Tub-Gal80^ts^; nSyb-Gal4.
