# Supplementary material for: A Drosophila model of mitochondrial disease phenotypic heterogeneity
Source: Biol Open. 2024 Feb 28;13(2):bio060278. doi: 10.1242/bio.060278 (PMC10924217; doi:10.1242/bio.060278)
Supplement: Supplementary information [file biolopen-13-060278-s1.pdf]

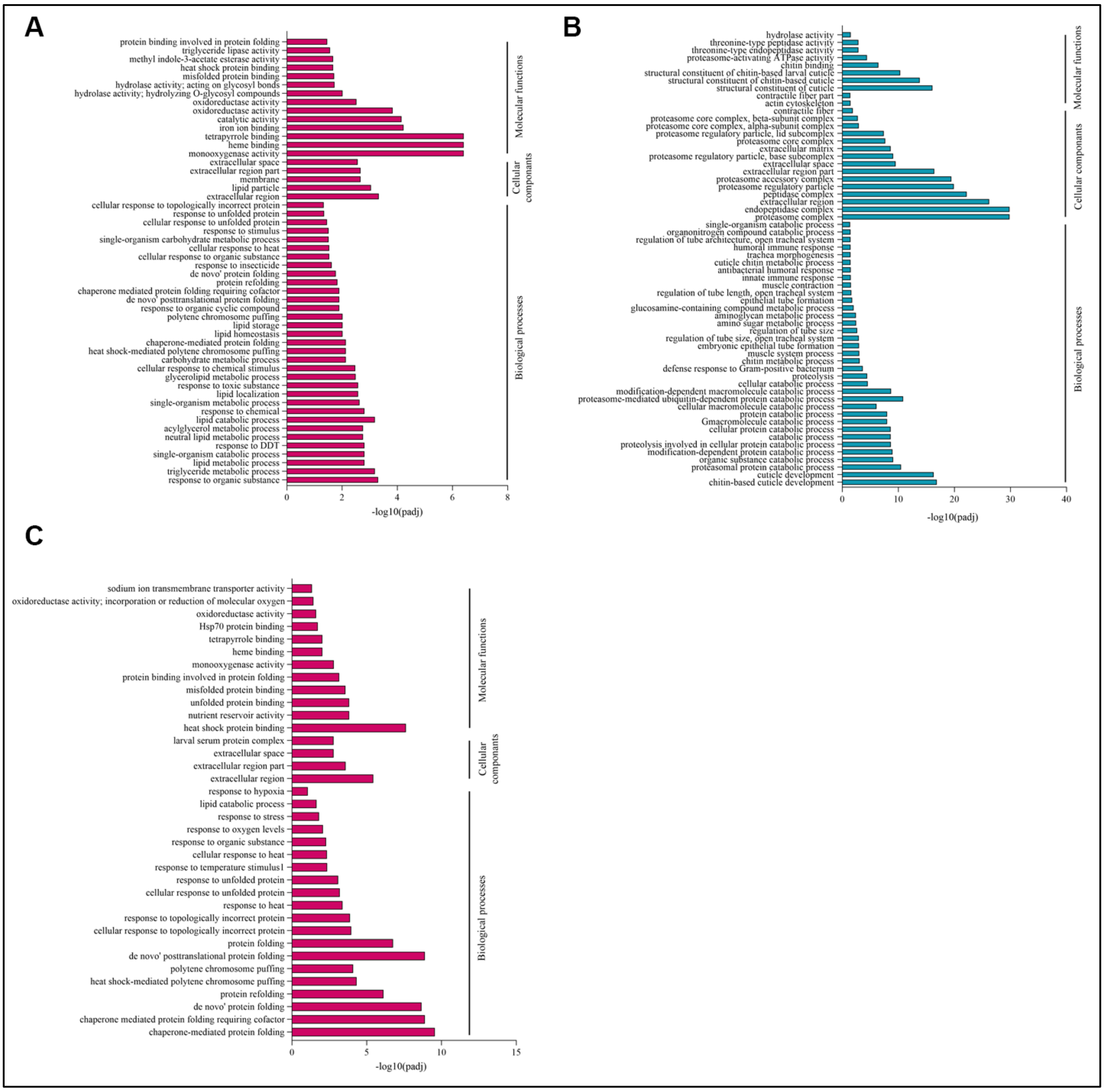

Fig. S1. GO analysis of the DEGs regulated by weak and strong ND-75 knockdown in neurons. (A) GO categories significantly enriched in genes with increased expression in head tissue from flies expressing ND-75^{KDweak}. (B) GO categories significantly enriched in genes with decreased expression in head tissue from flies expressing ND-75^{KDweak}. (C) GO categories significantly enriched in genes with increased expression in head tissue from flies expressing ND-75^{KDstrong}.

Biology Open • Supplementary information

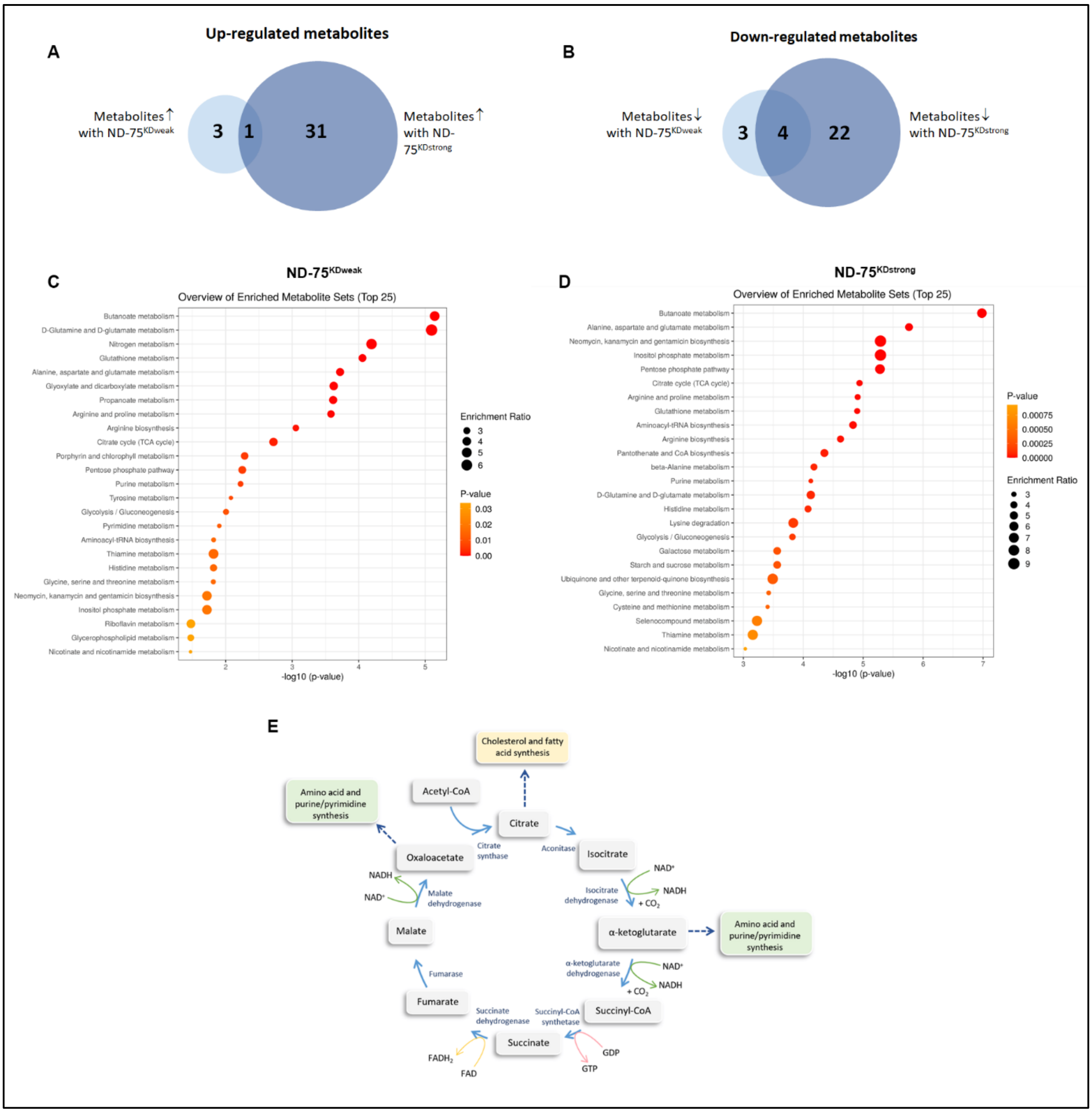

Fig. S2. Analysis of metabolomic changes caused by weak and strong ND-75 knockdown in neurons. (A, B) Numbers of metabolites significantly increased (A) and decreased (B) in ND-75^{KDweak} and ND-75^{KDstrong} head tissue and their overlap. (C, D) MSEA of metabolites with significantly altered levels ND-75^{KDweak} and ND-75^{KDstrong} head tissue. (E) Diagram of the TCA cycle.

Table S1. Genes with significantly increased expression in head tissue from flies with pan-neuronal ND-75^{KDweak} knockdown using Tub-Gal80^{ts}; nSyb-Gal4. Ranked by adjusted p value.

Available for download at
https://journals.biologists.com/bio/article-lookup/doi/10.1242/bio.060278#supplementary-data

Table S2. Genes with significantly decreased expression in head tissue from flies with pan-neuronal ND-75^{KDweak} knockdown using Tub-Gal80^{ts}; nSyb-Gal4. Ranked by adjusted p value.

Available for download at
https://journals.biologists.com/bio/article-lookup/doi/10.1242/bio.060278#supplementary-data

Table S3. Genes with significantly increased expression in head tissue from flies with pan-neuronal ND-75^{KDstrong} knockdown using Tub-Gal80^{ts}; nSyb-Gal4. Ranked by adjusted p value. MFS3 and Ldh highlighted in red.

Available for download at
https://journals.biologists.com/bio/article-lookup/doi/10.1242/bio.060278#supplementary-data

Table S4. Genes with significantly decreased expression in head tissue from flies with pan-neuronal ND-75^{KDstrong} knockdown using Tub-Gal80^{ts}; nSyb-Gal4. Ranked by adjusted p value.

Available for download at
https://journals.biologists.com/bio/article-lookup/doi/10.1242/bio.060278#supplementary-data

Biology Open • Supplementary information

Table S5. Metabolite levels, normalised to total ion count, from control, pan-neuronal ND-75^{KDweak} or ND-75^{KDstrong} fly head tissue using Tub-Gal80^{ts}; nSyb-Gal4. Colour of metabolite ID stands for: black = identity confirmed by standard or MS2, red = cannot separate, orange = identity not confirmed.

Available for download at
https://journals.biologists.com/bio/article-lookup/doi/10.1242/bio.060278#supplementary-data

Table S6. Metabolites with significantly altered levels in head tissue from flies with pan-neuronal ND-75^{KDweak} knockdown using Tub-Gal80^{ts}; nSyb-Gal4.

Available for download at
https://journals.biologists.com/bio/article-lookup/doi/10.1242/bio.060278#supplementary-data

Table S7. Metabolites with significantly altered levels in head tissue from flies with pan-neuronal ND-75^{KDstrong} knockdown using Tub-Gal80^{ts}; nSyb-Gal4.

Available for download at
https://journals.biologists.com/bio/article-lookup/doi/10.1242/bio.060278#supplementary-data

Table S8. Details of Drosophila strains used.

Stock	Full genotype	Source & stock number	Construct ID	Reference
w^{1118}	*w[1118];+/+;+/+;+/+*	BDSC 6326		
nSyb-Gal4	*y[1] w[*]; +/+; P{nSyb-GAL4.S}3; +/+*	BDSC 51635		
UAS-Dcr2;OK371-Gal4,UAS-CD8-GFP	*UAS-Dcr2;OK371-Gal4,UAS-CD8-GFP; +/+;+/+*	From Darren Williams, King's College London		[1]
Daughterless GeneSwitch-GAL4	*w[*]; P{da-GSGAL4.T};+/+;+/+*	Nazif Alic, University College London		[2]
Tubulin-$GAL80^{ts}$	*w[*]; P{w[+mC]=tubP-GAL80[ts]}10; TM2/TM6B, Tb[1];+/+*	BDSC 7108		
UAS-ND-75 RNAi [strong]	*y[1] sc[*] v[1] sev[21]; +/+;P{y[+t7.7] v[+t1.8]=TRiP.HMS00853}attP2;+/+*	BDSC 33910	HMS00853	[3]
UAS-ND-75 RNAi [weak]	*w[1118];* ND-$75^{KK108222}$; +/+; +/+	VDRC, v100733	KK108222	[4]
UAS-mitoGFP	*w[1118]; P{w[+mC]=UAS-mito-HA-GFP.AP}2/CyO;+/+;+/+*	BDSC 8442		[5]
Tub-Gal4	*w[1118]; +/+; Tub-Gal4/TM6B; +/+*	Helen McNeill, Washington University in St. Louis		

Biology Open • Supplementary information

References

1. Cagin U, Duncan OF, Gatt AP, Dionne MS, Sweeney ST, Bateman JM. Mitochondrial retrograde signaling regulates neuronal function. Proc Natl Acad Sci U S A. 2015;112(44):E6000-9. Epub 2015/10/23. doi: 10.1073/pnas.1505036112. PubMed PMID: 26489648.
2. Tricoire H, Battisti V, Trannoy S, Lasbleiz C, Pret AM, Monnier V. The steroid hormone receptor EcR finely modulates Drosophila lifespan during adulthood in a sex-specific manner. Mechanisms of ageing and development. 2009;130(8):547-52. Epub 20090530. doi: 10.1016/j.mad.2009.05.004. PubMed PMID: 19486910.
3. Perkins LA, Holderbaum L, Tao R, Hu Y, Sopko R, McCall K, et al. The Transgenic RNAi Project at Harvard Medical School: Resources and Validation. Genetics. 2015;201(3):843-52. Epub 20150828. doi: 10.1534/genetics.115.180208. PubMed PMID: 26320097; PubMed Central PMCID: PMCPMC4649654.
4. Dietzl G, Chen D, Schnorrer F, Su KC, Barinova Y, Fellner M, et al. A genome-wide transgenic RNAi library for conditional gene inactivation in Drosophila. Nature. 2007;448(7150):151-6. Epub 2007/07/13. doi: nature05954 [pii]
10.1038/nature05954. PubMed PMID: 17625558.
5. Horiuchi D, Barkus RV, Pilling AD, Gassman A, Saxton WM. APLIP1, a kinesin binding JIP-1/JNK scaffold protein, influences the axonal transport of both vesicles and mitochondria in Drosophila. Curr Biol. 2005;15(23):2137-41. doi: 10.1016/j.cub.2005.10.047. PubMed PMID: 16332540; PubMed Central PMCID: PMCPMC1532932.

Biology Open • Supplementary information
